# Ambient Moisture‐Induced Self Alignment of Polarization in Ferroelectric Hafnia

**DOI:** 10.1002/advs.202410354

**Published:** 2024-10-30

**Authors:** Lu‐Qi Wei, Zhao Guan, Wen‐Yi Tong, Wen‐Cheng Fan, Abliz Mattursun, Bin‐Bin Chen, Ping‐Hua Xiang, Genquan Han, Chun‐Gang Duan, Ni Zhong

**Affiliations:** ^1^ Key Laboratory of Polar Materials and Devices Ministry of Education Shanghai Center of Brain‐inspired Intelligent Materials and Devices East China Normal University Shanghai 200241 China; ^2^ Collaborative Innovation Center of Extreme Optics Shanxi University Taiyuan Shanxi 030006 China; ^3^ School of Microelectronics Xidian University Xi'an 710071 China

**Keywords:** ferroelectric materials, HfO_2_‐based, HZO, PFM, water

## Abstract

The discovery of nanoscale ferroelectricity in hafnia (HfO_2_) has paved the way for next generation high‐density, non‐volatile devices. Although the surface conditions of nanoscale HfO_2_ present one of the fundamental mechanism origins, the impact of external environment on HfO_2_ ferroelectricity remains unknown. In this study, the deleterious effect of ambient moisture is examined on the stability of ferroelectricity using Hf_0.5_Zr_0.5_O_2_ (HZO) films as a model system. It is found that the development of an intrinsic electric field due to the adsorption of atmospheric water molecules onto the film's surface significantly impairs the properties of domain retention and polarization stability. Nonetheless, vacuum heating efficiently counteracts the adverse effects of water adsorption, which restores the symmetric electrical characteristics and polarization stability. This work furnishes a novel perspective on previous extensive studies, demonstrating significant impact of surface water on HfO_2_‐based ferroelectrics, and establishes the design paradigm for the future evolution of HfO_2_‐based multifunctional electronic devices.

## Graphical abstract

In this paper, it is observed that polarization retention and stability of ferroelectric Hf_0.5_Zr_0.5_O_2_ (HZO) are significantly dependent on humidity. Elimination of absorbed water shows a significant effect in improving polarization stability and suppressing imprinting characteristics. This work provides a novel understanding of the relationship between surface electrochemistry and ferroelectricity in HfO_2_‐based ferroelectric materials.

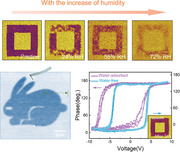

## 1 Introduction

Hafnia (HfO_2_) ferroelectric materials have drawn considerable interest over the past century due to their remarkable properties that challenge the conventional understanding of ferroelectric oxides.^[^ ^1^ ^]^ The localized electric dipoles in confined dimensions can be independently switched, making HfO_2_‐based ferroelectrics resistant to finite size effects (diminishing ferroelectricity due to size scaling) and a tendency to enhance polarization.^[^ ^2^ ^]^ This significantly promotes the development of next‐generation non‐volatile and high‐density devices. However, it remains controversial and underexplored whether intrinsic structural constraints or extrinsic oxygen vacancy migration mechanisms affect the stability of robust ferroelectricity in HfO_2_‐based ferroelectrics.^[^ ^3^ ^]^ One widely accepted explanation is lattice oxygen displacement in the polar orthorhombic phase (o‐phase).^[^ ^4^ ^]^ Interestingly, in comparison to the thermodynamically stable non‐polar monoclinic phase, the o‐phase is metastable.^[^ ^5^ ^]^

HfO_2_‐based ferroelectrics are sensitive to oxygen vacancies due to their inherently hydrophilic nature and charge trapping properties,^[^ ^6^ ^]^ therefore, many factors can influence the stabilization of the ferroelectric o‐phase, for example, clamping effects^[^ ^7^ ^]^ and electrode‐film interfaces.^[^ ^7^, ^8^ ^]^ In metal‐insulator‐metal (MIM) capacitor structures, preparing top and bottom electrodes before the annealing process can trigger the clamping effect and significantly increase the proportion of ferroelectric o‐phase.^[^ ^7^ ^]^ Meanwhile, if interfacial oxidation occurs between electrodes and films during annealing, oxygen vacancy density can be modified and thus remarkably enhances the stability of the ferroelectric o‐phase.^[^ ^9^ ^]^ Besides the effect of electrodes, recently, another important factor, film interface/surface conditions,^[^ ^8^, ^10^ ^]^ has been gaining increasing attention. A controlled gas environment allows adsorbates to play different roles in terms of providing the interfacial charges of bare HfO_2_‐based ferroelectric films. At ambient, highly inhomogeneous polarization is distributed, while oxygen migration can be promoted and screening charges type can be limited in reducing oxygen pressure atmosphere or vacuum. The vacuum condition is similar to the influence of conductive electrodes, stabilizing the ferroelectricity in HfO_2_‐based ferroelectrics.^[^ ^11^ ^]^

Considering water is unavoidable during fabrication or characterization, and the internal field of conventional ferroelectric materials is influenced by the surface water absorption, the interface polar discontinuity as well as the surface oxygen vacancies through annealing.^[^ ^12^ ^]^ So the effect of water adsorbates on the ferroelectric stability in HfO_2_‐based ferroelectric systems should be investigated further. In this paper, we demonstrate the negative effects of water in the Zr‐doped HfO_2_ (HZO) ferroelectric system under different humidity. An asymmetric polarization, as well as relaxation in HZO ferroelectric thin films, are observed by piezoresponse force microscopy (PFM), demonstrating that water molecule adsorbed on film surface can induce build‐in electric field and thus result in the imprint and hysteretic behaviors. Vacuum heating can remove the water molecules from the film surface and rebuild symmetric electric behaviors and the stability of ferroelectricity. This work provides a novel understanding of the relationship between surface electrochemistry and ferroelectricity in HfO_2_‐based ferroelectric materials. It offers a potential pathway for improving the performance of hafnium‐based ferroelectric devices.

## 2 Results

### 2.1 Characterization of Ferroelectricity

**Figure 1:**
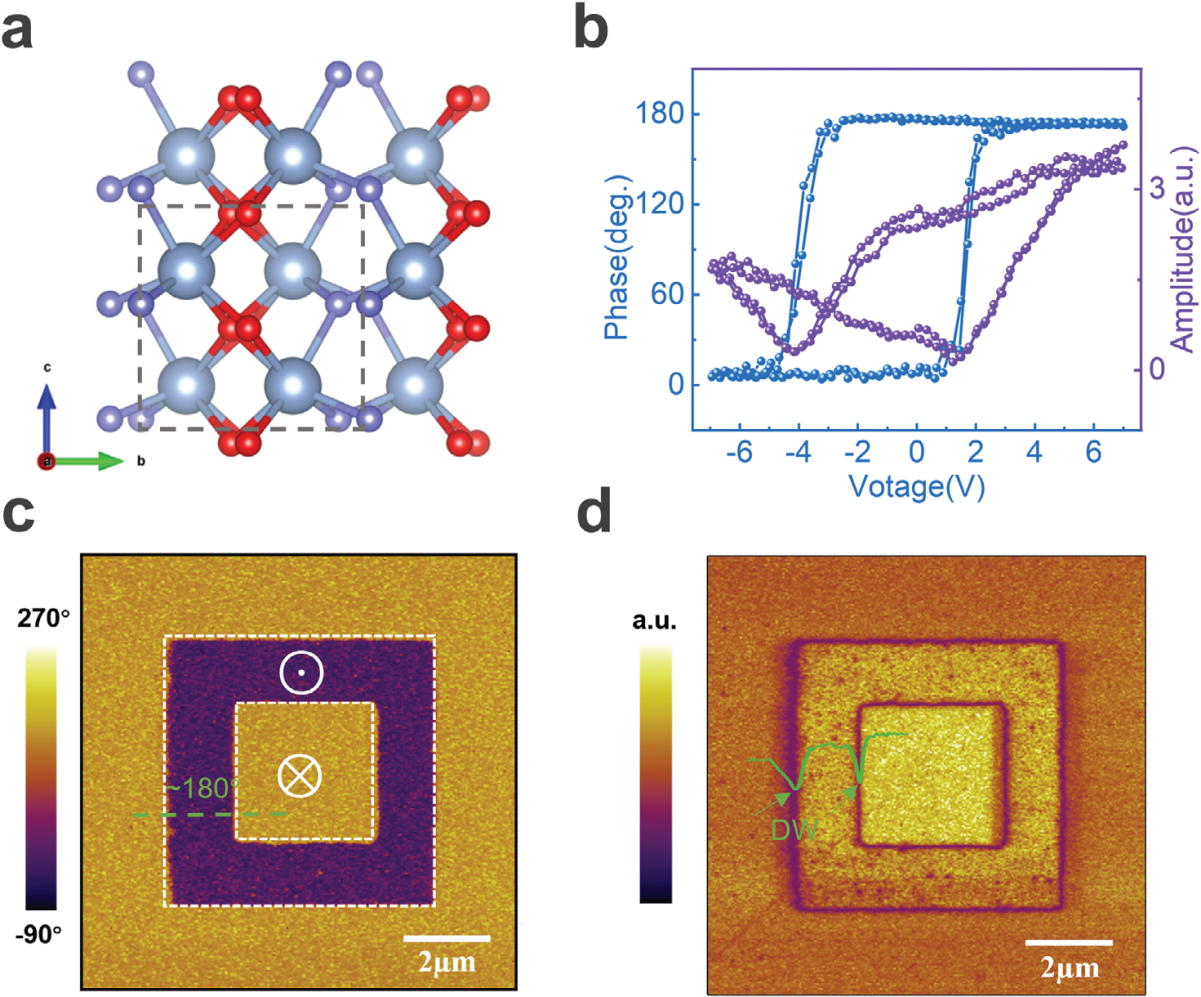
Ferroelectricity of HZO bare film. a) Crystal structure of the ferroelectric orthorhombic phase of hafnium oxide (Hafnium atoms in blue; Red is non‐polar oxygen and purple is polar oxygen atoms). b) Microscopic phase hysteresis loop and amplitude butterfly curves on HZO bare films by off‐field PFM. c,d) PFM phase c) and amplitude d) using the box‐in‐box pattern by scanning with the bias tip. The green dashed line c) shows the initial domains are the same as the positive voltage, with a phase difference of 180° from the negative voltage. The green line d) shows the domain wall between opposite polarizations.

The crystal structure is shown to have three‐coordinated polar oxygen separated by four‐coordinated no‐polar oxygen in the orthorhombic phase (**Figure** **1**a). The HZO films (≈12 nm) are deposited on a heavily doped silicon bottom electrode by atomic layer deposition and rapidly annealed under 550 °C for 30 s without the top electrode clamping. The Grazing incidence X‐ray diffraction indicates high quality of prepared HZO films and the topographic characterization reveals ultra‐flat film surfaces with a roughness of ≈0.22 nm (Figure S1, Supporting Information). Ferroelectric responses of the HZO thin films are characterized in Figure 1b, which indicates typical ferroelectric responses of a butterfly‐shaped amplitude and phase switching of ≈180°. Polarization switching and the initial polarization state are determined by applying an out‐of‐plane vertical electric field in the 10*10 µm^2^ range, using the box‐in‐box pattern. As shown in Figure 1c, a comparison of the two adjacent regions poled by the negative and positive voltages, respectively, reveals the downward initial polarization. The intrinsic ferroelectricity of HZO films is confirmed by the appearance of ferroelectric polarization with 180° phase contrast (Figure 1c; Figure S2, Supporting Information) and ferroelectric domain walls in PFM amplitude image (Figure 1d). Furthermore, a MIM capacitor structure is constructed by evaporating 50 nm Au/Cr square electrodes (25*25 µm^2^) on HZO films after rapid thermal annealing by lift‐off process (Figure S3a, Supporting Information). The hysteresis loop and current curve measured using the PUND (Positive‐up‐Negative‐down) pulse measurement (Figure S3b,c and Note S1, Supporting Information) shows that the film has a polarized downward imprint field with the coercive field of ≈ 4 V, and the value of remnant polarization P_r_ is ≈12.5 µC cm^−2^. The asymmetric behavior with a larger negative coercive field is consistent with that of microscopic PFM measurements.

### 2.2 Water‐Influenced Polarization Stability and Domain Retention

**Figure 2:**
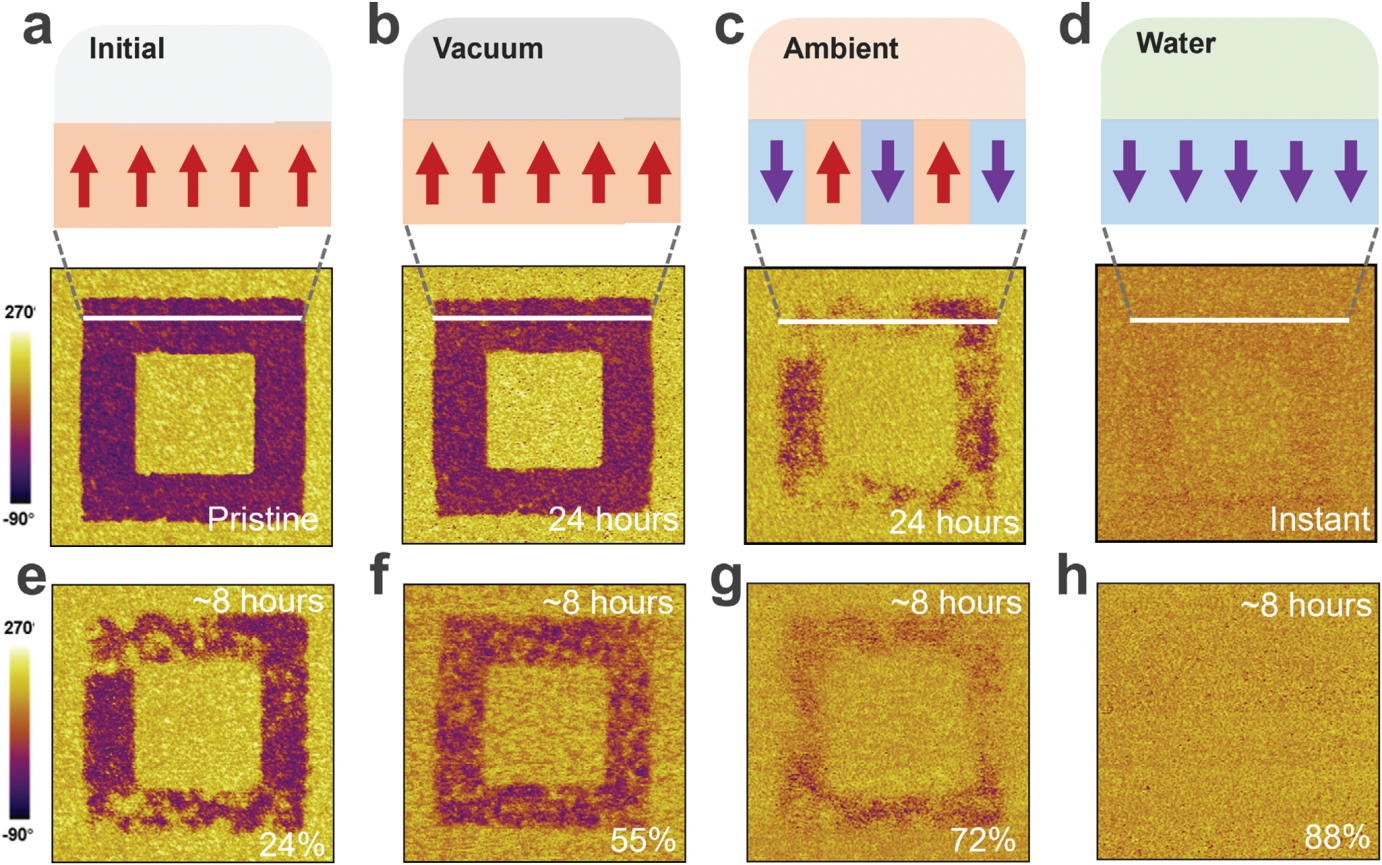
Polarization stability and domain retention affected by water molecule adsorption. a) Schematic diagrams PFM phase mappings of the pre‐polarized HZO thin films with tip bias of −9 V (8 µm wide violet box) and +9 V (4 µm wide yellow box). Note, image size is 10 µm; Red (purple) arrows indicate upward (downward) polarization. b,c) Schematic diagrams and PFM phase mappings of the pre‐polarized HZO film in b after keeping under vacuum b) and ambient (23 °C, 101 kPa and the relative humidity of 21%; c) for 24 h. d) Schematic diagrams PFM phase mappings of the pre‐polarized HZO film after covered with water and immediately removed. e–h) PFM phase mappings of pre‐polarized HZO films after maintaining them in different environments: 23 °C, 101 kPa and the relative humidity of 24% e), 55% f), 72% g), and 88% h) for 8 h, respectively.

The retention characteristics of ferroelectric domains were studied, by comparing the PFM response of domains directly after poling to the ones maintained for 24 h in the ambient and high vacuum (Note S2, Supporting Information). First, **Figure** **2**a as an example displays the initial poling processes of HZO film. Then, we pole a new location each time when change the different environments. Interestingly, the upward pre‐polarized ferroelectric domains in ambient have generally flipped downward, while those in high‐vacuum remained stable, as shown in Figure 2b,c. Notably, when exposed to deionized water for several seconds, the upward polarization domain patterns completely disappear (Figure 2d), suggesting the polarization switch from upward to downward. The corresponding domain walls of the pre‐polarized regions are shown in Figure S4 (Supporting Information). This indicates that the external environment especially for polar water molecules in the atmosphere, severely impacts the stability of the HZO thin film ferroelectric properties. Therefore, pre‐polarized HZO films were preserved in various humidity conditions ranging from 24% to 88% for 8 h (Note S2, Supporting Information). It is observed that the upward domain stability of HZO films is strongly dependent on environmental water contents (relative humidity). As humidity increases, the area of upwardly polarized domains gradually diminishes and flips downward, whereas downwardly polarized domains remain stable (Figure 2e–h). The trend of the percentage of upwardly polarized domains with increasing relative humidity and time is shown in Figure S5 (Supporting Information). This suggests that water molecule adsorption on film surfaces induces ferroelectric domains to spontaneously flip to a downward polarization orientation.

### 2.3 Adsorption of Water Molecules

**Figure 3:**
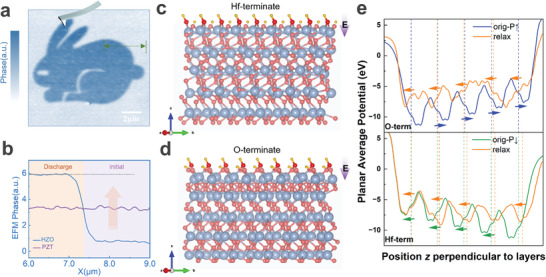
Build‐in electric field induced by ordered adsorption of water molecules. a) Corresponding EFM phase mappings after contact scanning of a specific rabbit pattern region on the surface of the HZO film using a grounded needle tip. b) The EFM phase difference corresponds to the green dashed region of a) and the PZT in the same region. c,d) Schematic diagram of water molecule adsorption on the surface of ferroelectric orthorhombic HfO_2_ structure with c) Hf‐terminate and d) O‐terminate. The purple arrows represent the direction of the additional electric field generated by the water molecules. e) The planar average of the electrostatic potential energy along the z direction in O‐terminate and Hf terminate by theoretical simulation. The directions of local electric dipoles are indicated by arrows, with respect to Hf‐located planes marked with dashed lines. The left sides correspond to downward directions (−z) in our adsorption models.

We further explore the relationship between surface water adsorption and ferroelectric polarization by electrostatic force microscopy (EFM). EFM with nanoscale resolution allows for the determination of numerous electrical properties of the sample surface, such as localized charges and surface potential distribution, using the potential difference between the conductive needle tip and the film.^[^ ^13^ ^]^ In addition, the contact mode can be utilized to achieve a discharge process in which surface adsorbents and impurity particles are effectively swept away when the tip is scanned in contact with the film surface. When the tip is scanned in grounded mode, the surface charge can be exported to obtain an electrically neutral surface. The schematic of EFM is given in Figure S6 and Note S3 (Supporting Information). As shown in **Figure** **3**a, the grounded tip is employed to perform contact scanning on the surface of the HZO film in a specific rabbit‐patterned area to discharge the surface adsorbates such as water molecules, H^+^, OH^−^, and so on. There is a significant difference between the discharged and initial regions. In Figure 3b, the line profile denoted by the green dash in Figure 3a is extracted. In comparison to the untreated region, the region where discharge was performed with the grounded tip showed an elevated EFM phase, implying an increased attraction force between the probe and the film. Considering the complemental dc bias applied to the tip during the test is +3 V, the surface potential in the discharging region is reduced relative to the initial region. In contrast, the same treatment was applied to the conventional ferroelectric material Pb(Zr, Ti)O_3_ (PZT) with a single polarization. No change in the EFM phase was observed, which is different from the results of HZO, as shown in Figure S7 (Supporting Information), indicating that the surface of the HZO film displays different electrochemical responses to water molecule adsorption and dissociation. Besides, the EFM phase variation of the film surface over time in a low‐humidity environment was monitored (Figure S8, Supporting Information). The EFM phase difference between the discharge and the initial region decreases with time, suggesting that the water adsorption and dissociation could occur spontaneously on the surface of the HZO film before it can be restored to a stable electrochemical surface state.

Density functional theory is employed to investigate the interaction between HfO_2_ films and H_2_O in air at the atomic level. To avoid complicated calculations, we simplify the model by considering a 1 × 1 × 5 HfO_2_ slab with an adsorbed H_2_O molecule. For the HfO_2_ slab, upward and downward polarization cases terminated by Hf^+^, as well as O^2−^‐terminated surfaces with and without oxygen vacancies are taken into account. The polarization of H_2_O molecules is initially set to be pointing downward. As illustrated in Figures 3c,d, and Figure S9a,b (Supporting Information), the above H_2_O molecule moves toward the beneath ferroelectric film regardless of the surface charge and polarization directions of HfO_2_. Interestingly, H_2_O molecules always turn upward polarized after fully relaxing, even while dissociating to H^+^ and OH^−^ ions, indicating a built‐in electric field pointing downward. The large electric field generated by O‐H bonds in water,^[^ ^14^ ^]^ which is powerful enough to reverse the polarization of HfO_2_. As expected, our theoretical calculations of electrostatic potential energy Figure 3e and Figure S9c (Supporting Information) confirm the significant effect of water in the air on the downward polarization of the HfO_2_ slab (Note S4 for more details, Supporting Information).

### 2.4 Elimination of Water‐Induced Imprint Effects

**Figure 4:**
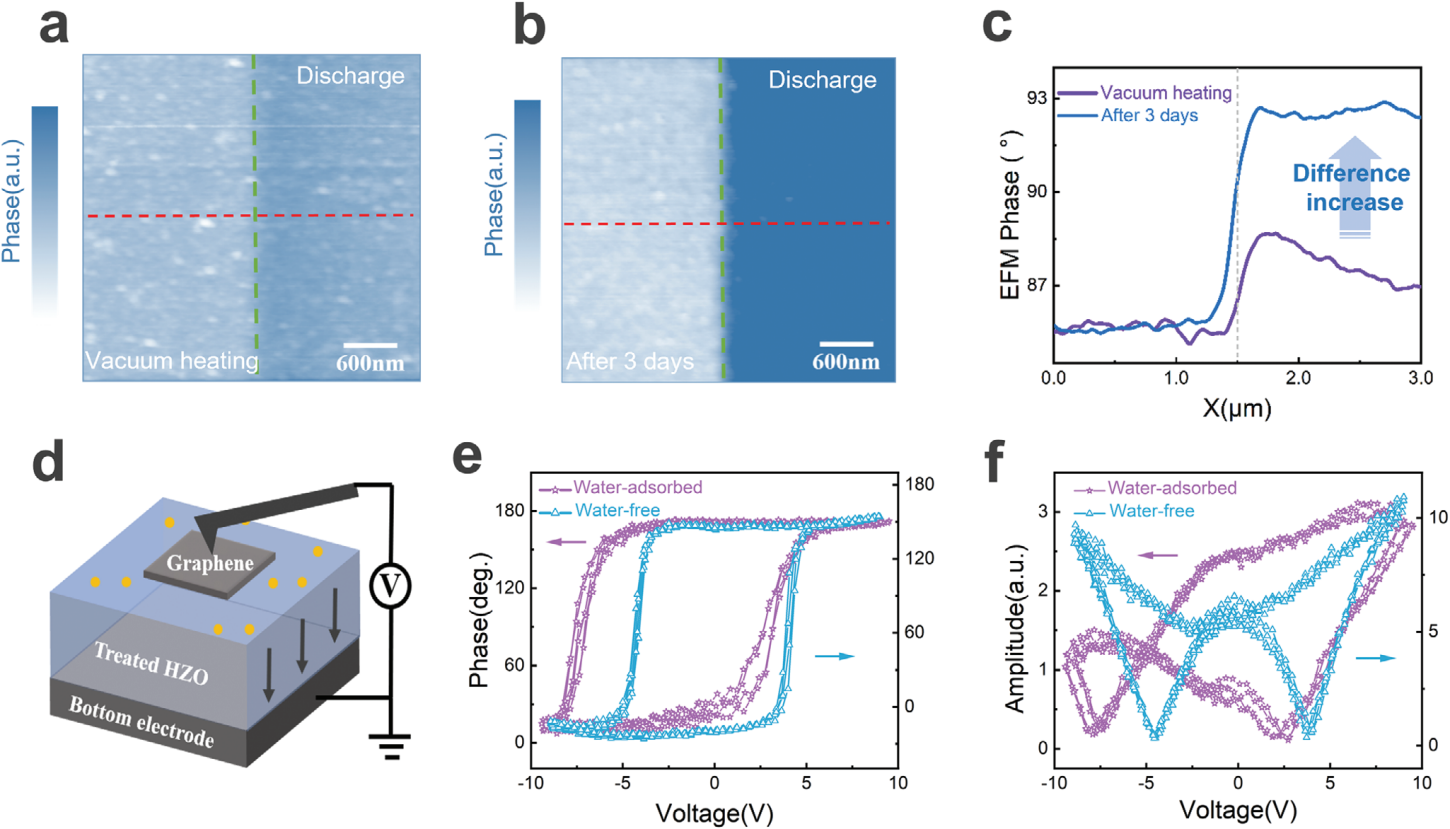
Symmetrical coercive field through vacuum heating treatment. a,b) EFM phase mappings corresponding to a discharge scanning after vacuum heating treatment at 110 °C 10^−3^ mbarr for 2 h a) and three days b) in the atmosphere. c) EFM phase corresponding to the initial and discharge regions in a,b). d) Schematic showing the graphene/(water)/HZO/silicon capacitor structure. e,f) The off‐field PFM phase e) and amplitude f) hysteresis loops measured in the graphene/(water)/HZO/silicon capacitor structure.

The HZO films are subjected to vacuum heating for 2 h at 110 °C and 10^−3^ mbarr to minimize the influence of water adsorption on the electrochemical surface state of HfO_2_‐based ferroelectric films. The surface potential after discharge was compared to vacuum heating and after 3 days in air (**Figure** **4**a), resulting in significantly reduced EFM phase differences (Figure 4b). The findings show that vacuum heating can modify the electrochemical surface state of HZO thin films. In Figure 4c, the EFM phase of the corresponding discharge region versus the initial region for the HZO film surface water‐free and water‐absorbed were depicted, as labeled by red dash in Figure 4a,b. The built‐in electric field constructed decreases dramatically when surface water molecules are removed via vacuum heating. To visualize the surface water build‐in electric field, 10 nm thick graphene flakes were mechanically exfoliated and transferred onto the water‐free and water‐absorbed HZO surface to form the capacitor structure of graphene/(water)/HZO/silicon (Figure 4d). Figure 4e,f illustrates the off‐field PFM phase hysteresis loops as well as butterfly curves, and the differential loops (on‐field minus off‐field) indicate almost no electrostatic artifacts during switching (Figure S11, Supporting Information).^[^ ^15^ ^]^ Interestingly, vacuum‐heated HZO films exhibit symmetric hysteresis, whereas water‐absorbed films have asymmetric imprinting characteristics. It is important to note that the water‐absorbed films are samples that have been vacuum heated and then exposed to the atmosphere (the relative humidity of 24% and room temperature) for 3 days, which eliminates the impact of structural changes inside the HZO on the imprint field. As shown in Figure S10 (Supporting Information), the pristine HfO_2_ is characterized by the symmetric double well suggesting an equal polarity of this system. In the water‐adsorbed HfO_2_, the positive side (upward polarization) of the double well is higher in energy than the negative side (downward polarization), making the energetically favored ferroelectric state with downward polarization. As a result, the formation of asymmetric imprinting behavior can be attributed primarily to the built‐in electric field, which increases the internal downward electric field and causes the ferroelectric polarization to be more inclined toward down.

## 3 Conclusion

In summary, this work demonstrates the atmosphere humility dependence of polarization stability in HZO thin films. With increase of humidity, the upward polarization retention decreases. The upward‐polarized domains of ferroelectrics could be well switched by a simple water treatment. Our findings may provide an explanation for why most bare HZO films consistently exhibit a single downward polarization direction in previous works. EFM results confirm that water molecule adsorption on the surface of HZO films alters the electrochemical surface state. It is proposed that the adsorption of water molecules on the film's surface generates a built‐in electric field directed toward the bottom electrode, affecting both the stability and retention properties of HZO ferroelectric domains. The observed water‐adsorbed phenomena contribute to the build‐in field, resulting in imprinting effects, which are detrimental to the practical application of HfO_2_‐based ferroelectrics. More importantly, we propose a simple and effective method: vacuum treatment to eliminate surface water adsorption, which can be maintained by electrode coverage. This approach yields highly symmetric phase hysteresis loops. This provides a very interesting path to optimize the preparation process as well as the functional design of memory and logic devices.

## 4 Experimental Section

### Fabrication

HZO thin films were grown on heavily doped silicon substrates by atomic layer deposition. First, the substrate was sonicated for 10 min using acetone, isopropanol, and deionized water sequentially to avoid the presence of surface impurities affecting the deposition quality of the films. Then the surface natural oxides were removed using aqueous hydrofluoric acid (HF/H_2_O = 1:50). After that, 12 nm thick HZO films were grown at 280 °C using [(CH_3_)_2_N]_4_Hf (TDMAHf), [(CH_3_)_2_N]_4_Zr (TDMAZr) and H_2_O as Hf, Zr precursors and oxygen source, respectively. Finally, it was crystallized by rapid thermal annealing at 550 °C for 30 s under a nitrogen atmosphere to obtain the doped hafnium oxide thin films with ferroelectric orthorhombic phase. The gate electrode was heavily doped silicon, The source and drain metal contacts were fabricated by a lift‐off process using direct write “Maskless” lithography and thermal evaporation by pre‐evaporation of 40 nm Au/10 nm Cr on hafnium oxide films with a channel width of 5 µm and channel length of 15 µm. The few‐layer MoS_2_ (2–5 nm thickness) are mechanically exfoliated and transferred to the region between source and drain contacts

### Piezoresponse Force Microscopy

Ferroelectric domains and piezoresponse hysteresis loops were performed using a DART PFM (Cypher S, Asylum Research). NSC18 conductive tip from Micromash with a force constant of 2.8 N m^−1^ was driven with a 0.5 V ac voltage under a contact resonant frequency of ≈320 kHz during all the PFM measurements. The Lithography mode was used to apply dc voltage by specific pattern.

### Electrostatic Force Microscopy

The EFM measurements also used Asylum Research Cypher S system and this was done in double pass. During the measurements, maintain a 3 V dc voltage applied to the tip of the needle and elevate it by 30 nm during the double pass scan.

### Calculations

The DFT calculations were performed using the accurate full‐potential projector augmented wave method,^[^ ^16^ ^]^ as implemented in the Vienna ab initio Simulation Package (VASP).^[^ ^17^ ^]^ The exchange‐correlation potential was treated in the PBE (PerdewBurke–Ernzerhof) form^[^ ^18^ ^]^ of the generalized gradient approximation with a kinetic energy cutoff of 600 eV. A 9 × 9 × 1 Monkhorst‐Pack *k*‐point mesh in geometry optimizations was considered. The convergence criterion for the electronic energy was 10^6^ eV and the structures were relaxed until the Hellmann–Feynman forces on each atom are <1 meV Å^−1^. The in‐plane lattice constant was constrained to the optimized bulk HfO_2_ lattice constant *a* = 5.259 Å; *b* = 5.048 Å, in good agreement with the experimental data.^[^ ^4^ ^]^ Five unit cell layers were used to emulate the HfO_2_ thin film with O^2−^ and Hf^+^ terminal surfaces. Further checks using a ten‐unit‐cell‐layer HfO_2_ slab confirm the convergence of our theoretical results from geometric and electronic points of view. To prevent spurious electrostatic effects in the treatment of asymmetric structures with periodic boundary conditions, the dipole correction^[^ ^19^ ^]^ was applied in all simulations. A vacuum region with a thickness of ≈20 Å along the *z*‐axis was included between repeated slabs to eliminate the spurious slab‐slab interactions.

## Conflict of Interest

The authors declare no conflict of interest.

## Supporting information



Supporting Information

## Data Availability

The data that support the findings of this study are available from the corresponding author upon reasonable request.
