# Supplementary material for: Ambient Moisture‐Induced Self Alignment of Polarization in Ferroelectric Hafnia
Source: Adv Sci (Weinh). 2024 Oct 30;11(48):2410354. doi: 10.1002/advs.202410354 (PMC11672260; doi:10.1002/advs.202410354)
Supplement: Supplementary file 1 — Supporting Information [file ADVS-11-2410354-s001.docx]

Supporting Information

**Ambient Moisture-induced Self Alignment of Polarization in Ferroelectric Hafnia**

*Lu-Qi Wei^1,†^, Zhao Guan^1,†^, Wen-Yi Tong^1,†^, Wen-Cheng Fan^1^, Abliz Mattursun^1^, Bin-Bin Chen^1, 3^, Ping-Hua Xiang^1, 3*^, Genquan Han^2^, Chun-Gang Duan^1, 3^&Ni Zhong^1,3*^*

**AFFILIATIONS**

^1^Key Laboratory of Polar Materials and Devices, Ministry of Education, Shanghai Center of Brain-inspired Intelligent Materials and Devices, East China Normal University, Shanghai, 200241, China

^2^School of Microelectronics, Xidian University, Xi’an, 710071, China

^3^Collaborative Innovation Center of Extreme Optics, Shanxi University, Taiyuan, Shanxi, 030006, China*.*

^†^These authors contributed equally to this work.

^*^Correspondence and requests for materials should be addressed to P.-H.X. (email: [phxiang@ee.ecnu.edu.cn](mailto:phxiang@ee.ecnu.edu.cn)) or N.Z. (email: nzhong@ee.ecnu.edu.cn)


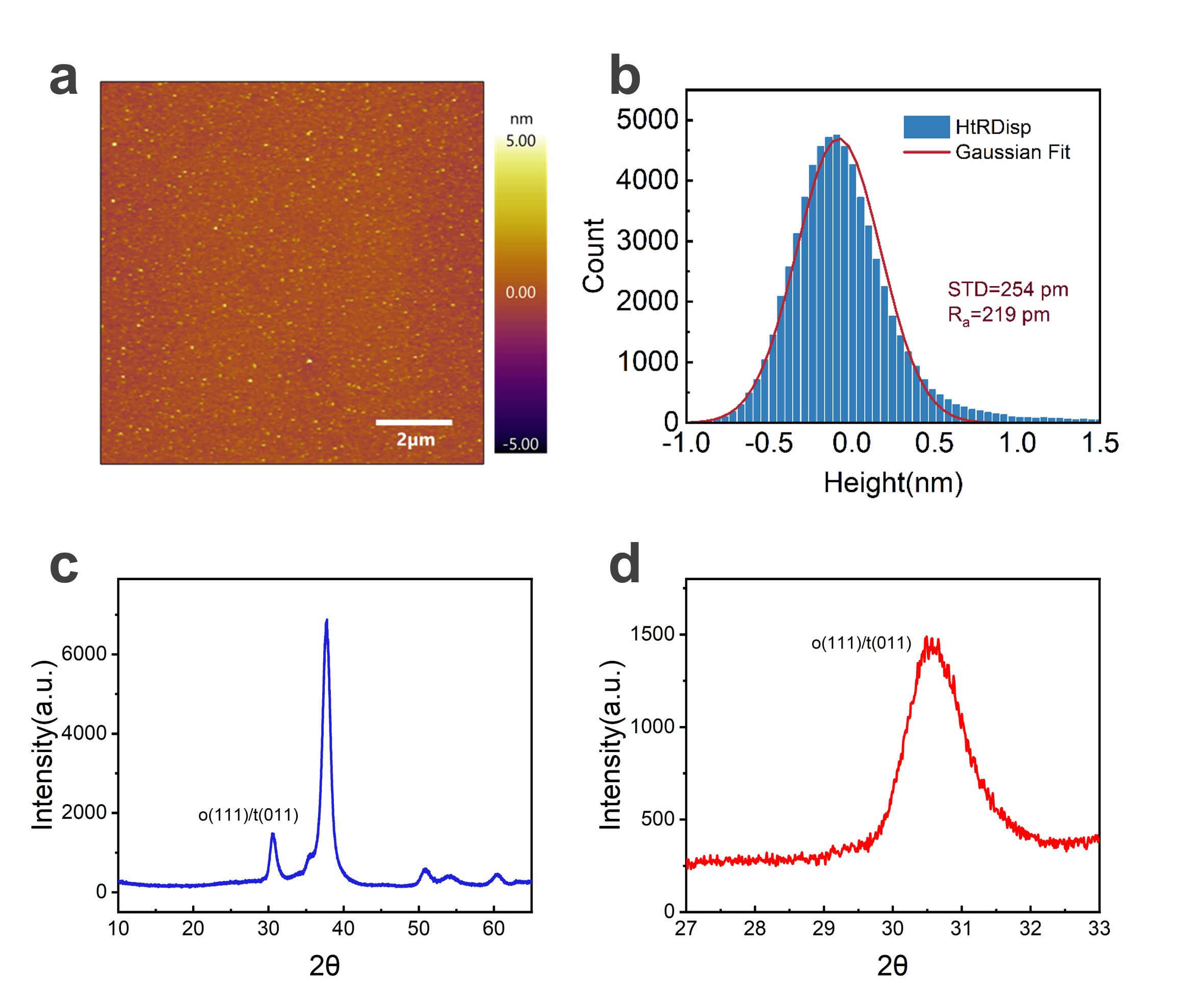


**Figure S1. Topography and roughness characterization.** (a) Surface topography of HZO thin films prepared by atomic layer deposition method, scale bar is 2 μm. (b) The histogram corresponding to the topography shown in (a) shows a standard deviation (STD) of 254 pm by Gaussian fitting. Where the arithmetic mean roughness (R_a_) of the film is 219 pm, indicating that ultra-flat and clean-surface HZO ferroelectric films were successfully prepared. (c, d) The Grazing incidence X-ray diffraction for HZO thin film. 2θ, the angle between the transmitted beam and reflected beam.


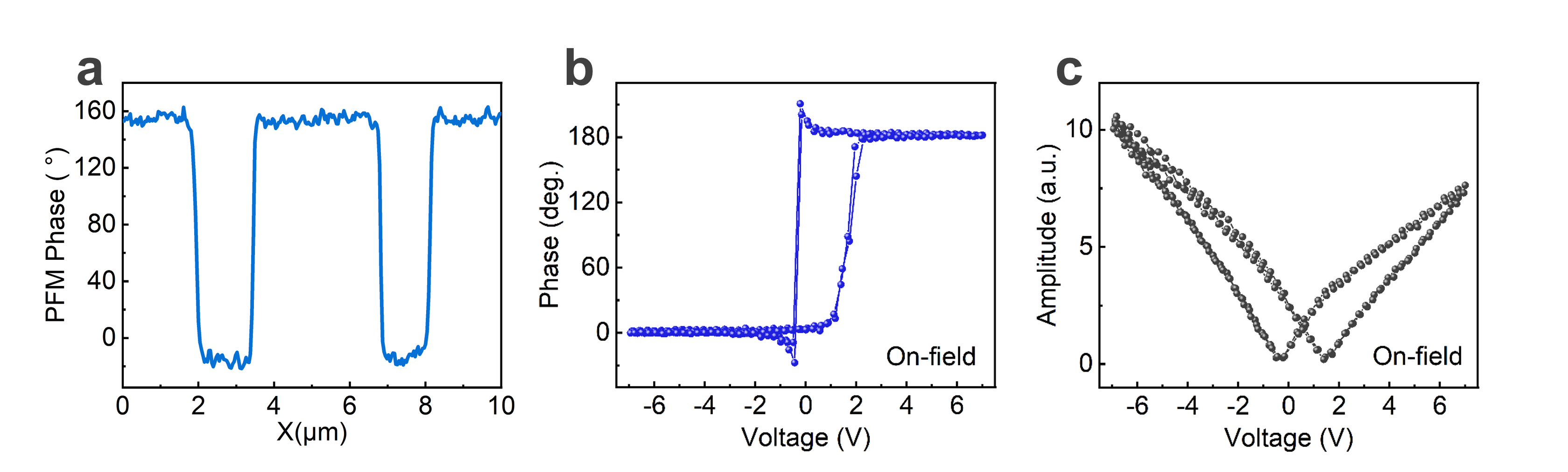


**Figure S2. On-field PFM loops.** (a) The PFM corresponding phase signals showing 180deg contrast in Figure 1c. (b, c) The PFM hysteresis loop (b) obtained and amplitude butterfly curves (c) using on-field PFM method for Figure 1b.


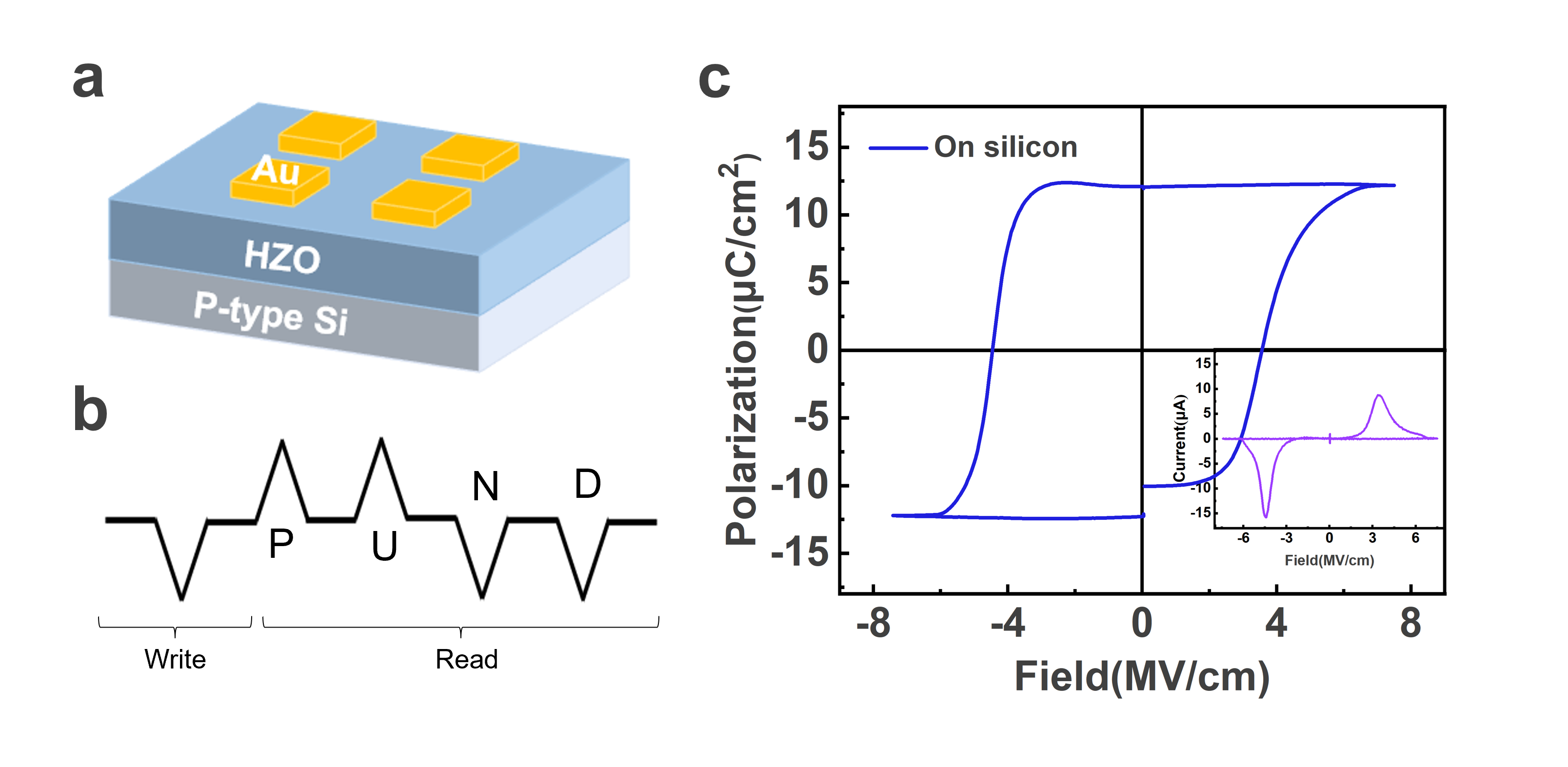


**Figure S3. Macroscopic ferroelectric characterization.** (a) Schematic structure of the prepared metal-HZO-metal capacitor. The top electrode is a 50 nm thick, 25 µm wide square electrode prepared by thermal evaporation equipment and the bottom electrode is p-type heavily doped silicon. (b) A specific pulse waveform applied during the PUND measurement. (c) Macroscopic P−E loops and current curve obtained by PUND pulses.


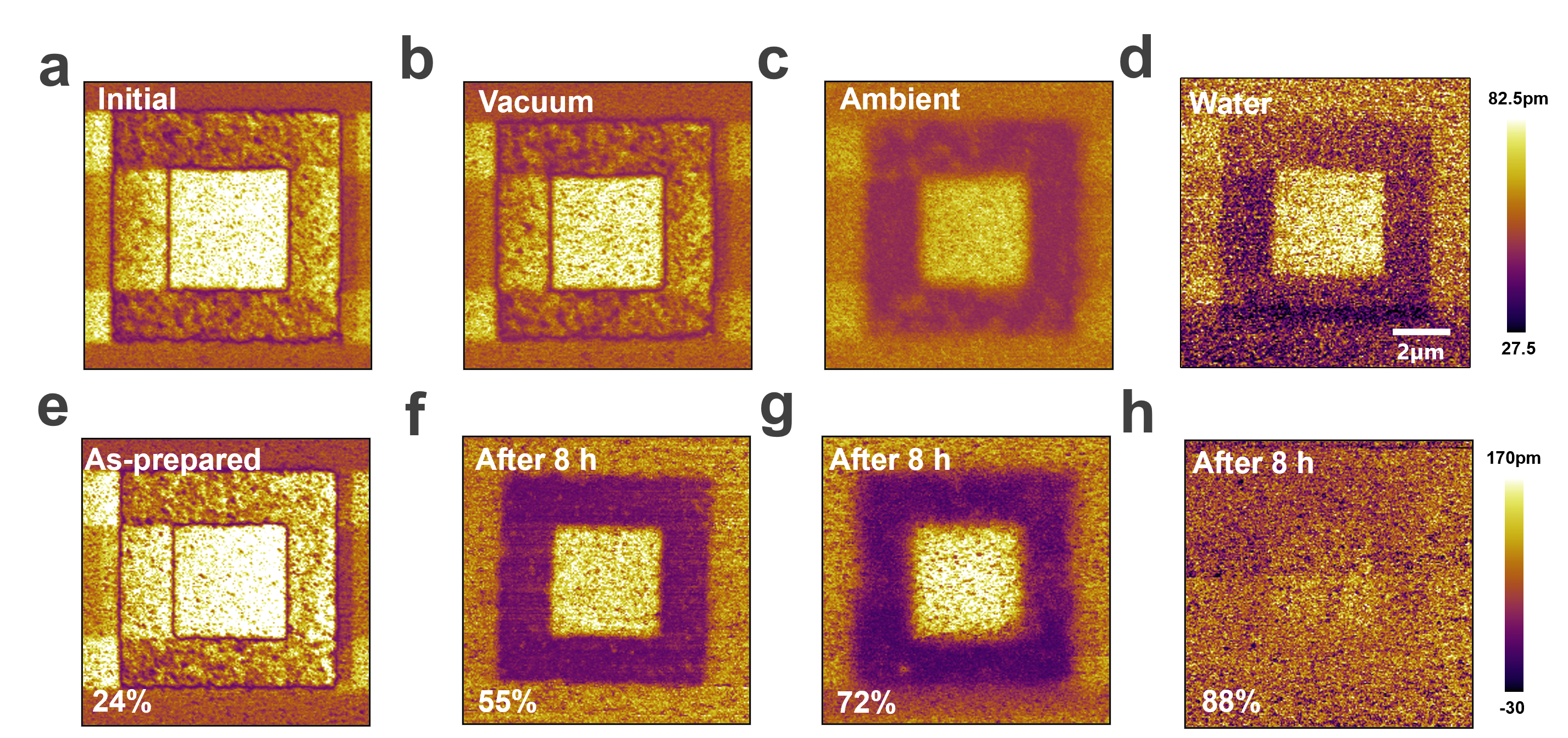


**Figure S4.** **PFM amplitude retention in different environments and humidities.** (a) PFM amplitude mapping of the box-in-box pattern (no voltage, -8V, +8V) through PFM tip bias. (b-d) PFM amplitude mappings in vacuum(24h), ambient(24h) and water(instant), respectively. (e-h) PFM amplitude mappings of pre-polarized HZO films after maintaining them in relative humidity of 24%, 55% and 88% for 8h.


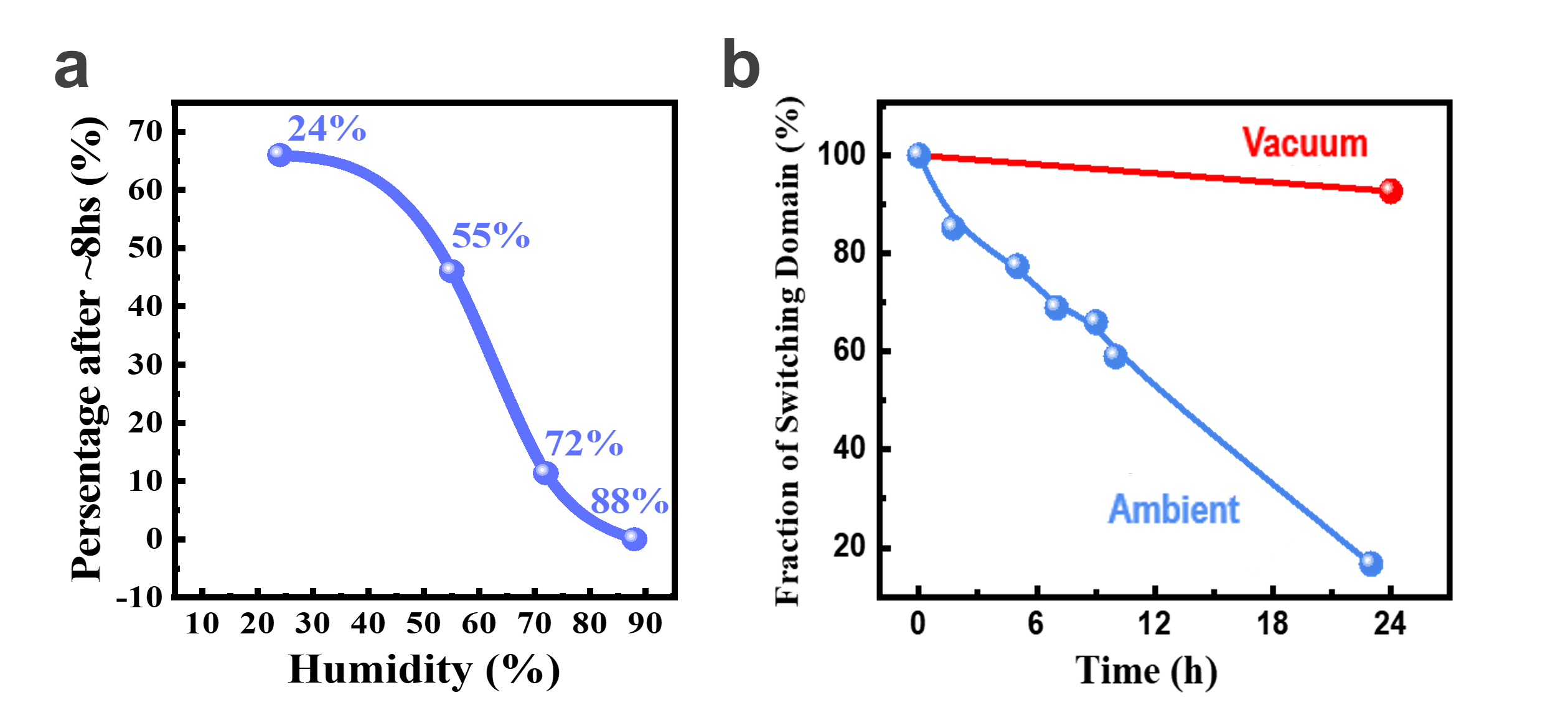


**Figure S5.** **Stability of upwardly polarized domains affected by water molecule adsorption.** (a) Variation of the percentage of upwardly polarized domains as a function of relative humidity. (b) Time-dependent percentage of upwardly polarized domains in HZO films placed in ambient and vacuum.


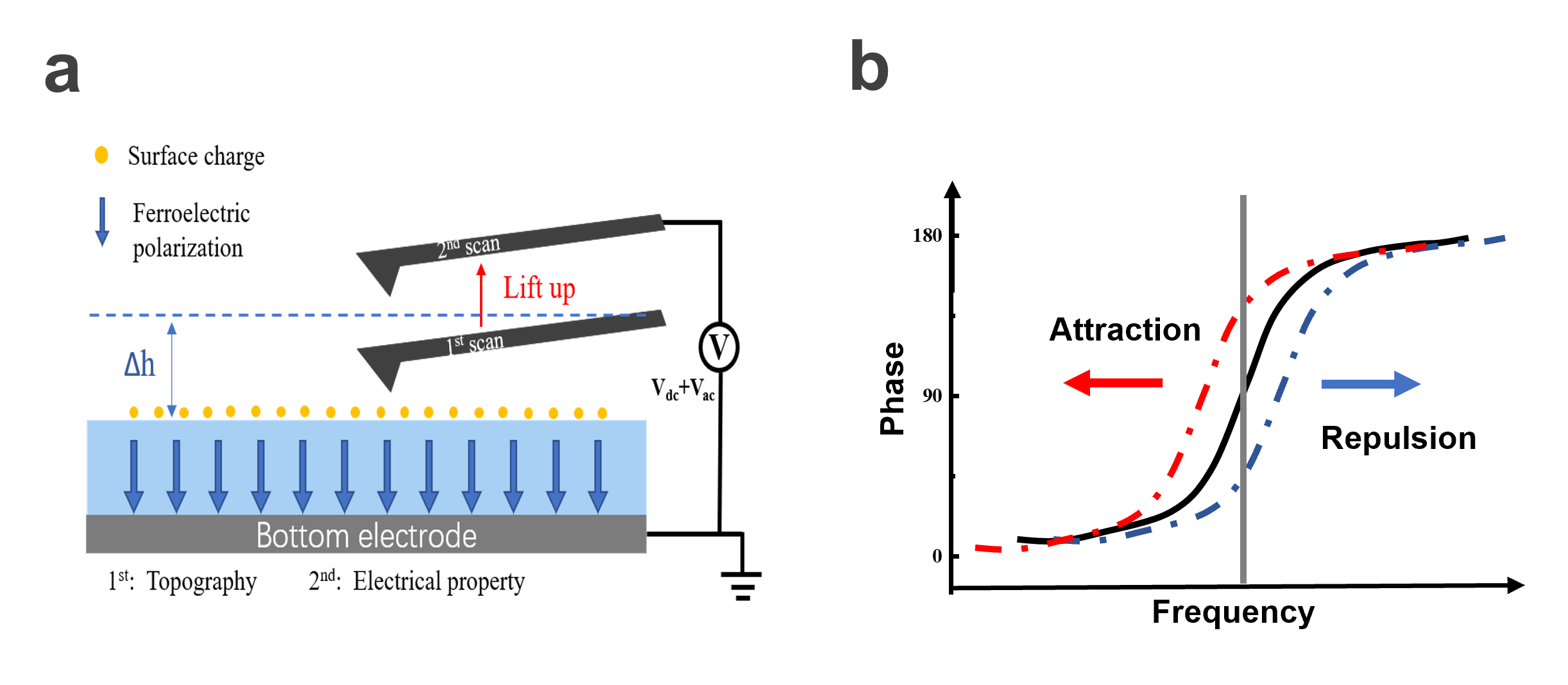


**Figure S6.** **Electrostatic force microscopy (EFM) measurements.** (a) Schematic diagram of EFM measurement principle. (b) Schematic diagram of the phase shifts induced by the probe cantilever under the action of long-range electrostatic forces.


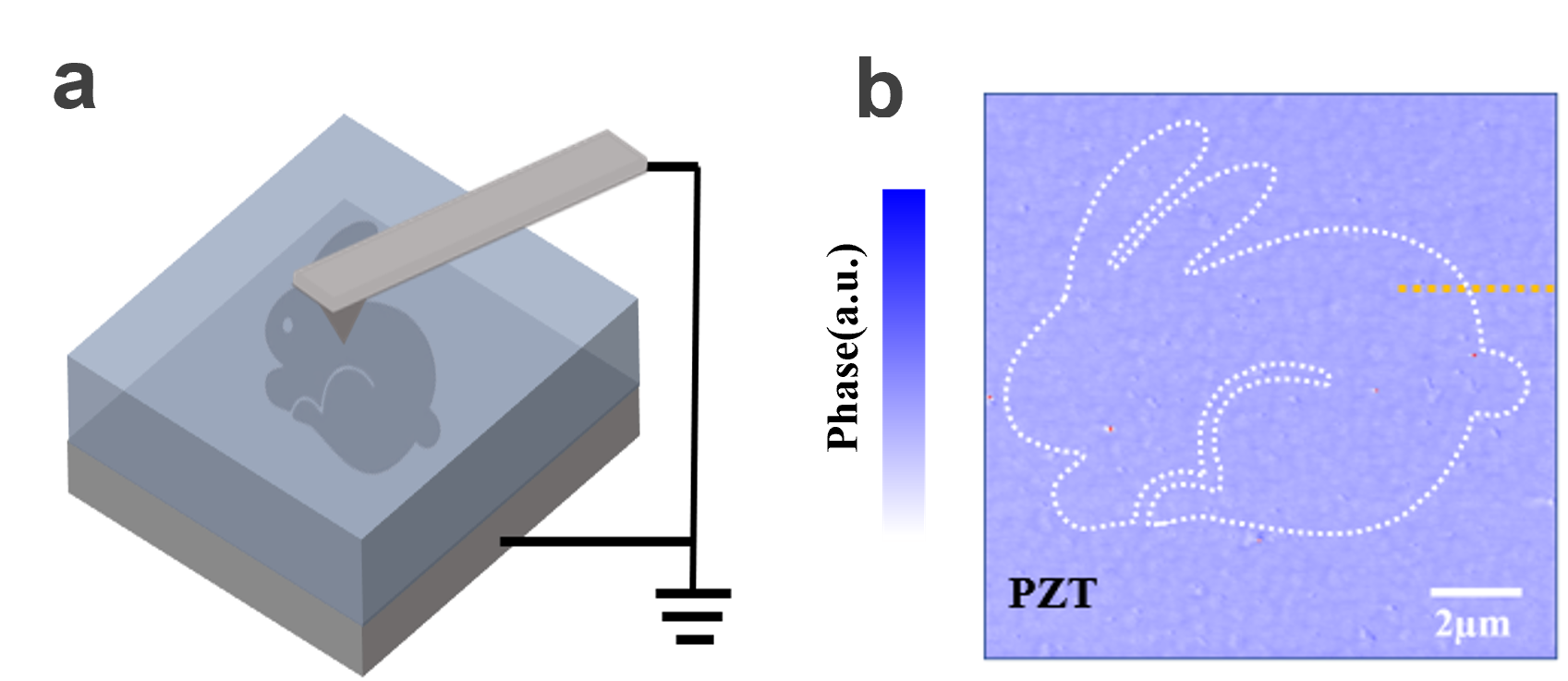


**Figure S7.** **Effect of water molecule adsorption on conventional ferroelectric materials.** (a) Schematic diagram of discharge process, where the tip of the needle is grounded and contact scanning is performed in the rabbit-shaped region of the figure. (b) Contact scanning on the PZT surface using the discharge process shown in (a) and characterizing a slightly larger range of EFM did not reveal any significant phase difference, suggesting that the surface adsorption of water molecules has a different effect on hafnium-based ferroelectrics and the conventional ferroelectric material (PZT).


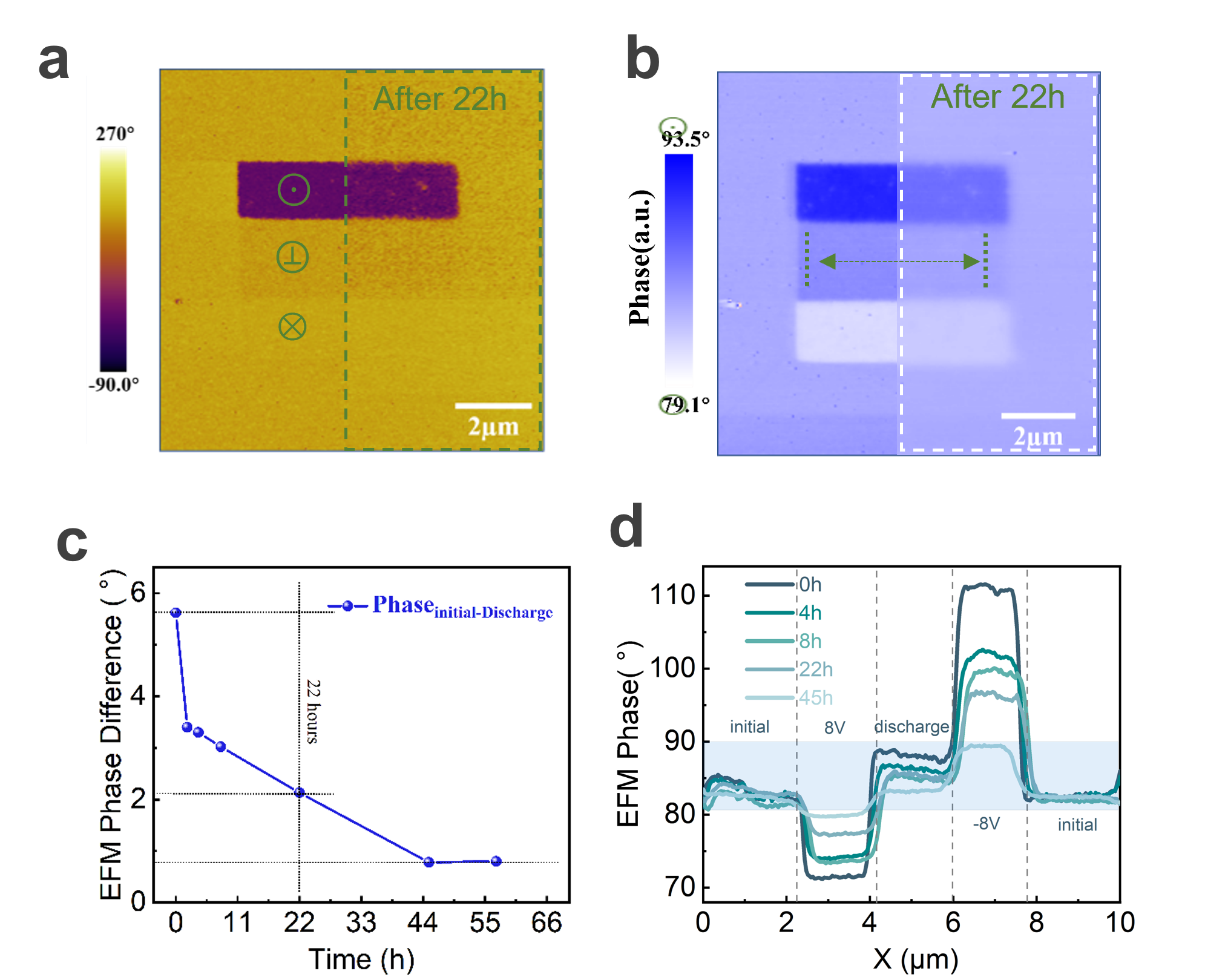


**Figure S8.** **Spontaneous water molecule adsorption and dissociation processes.** (a) PFM phase after 0h and 22h contact scanning on the film surface using a charged (±8V) probe and a grounded probe, respectively. Since only a small portion of the polarization up domains flip in low humidity environments, there is no change in the grounded and polarization flipped down regions. (a) Corresponding EFM phase change in (a). (c) Line graph of the EFM phase difference between the discharge region and the initial region as a function of time. (d) Relationship between the EFM phases in the applied positive and negative voltage regions as well as in the discharge region as a function of time tends to converge to the EFM phase in the initial region, which indicates that not only do the water molecules in the air shield the injected charge, but also that water molecules tend to adsorb and dissociate on the HZO surface to achieve a stable electrochemical surface state.


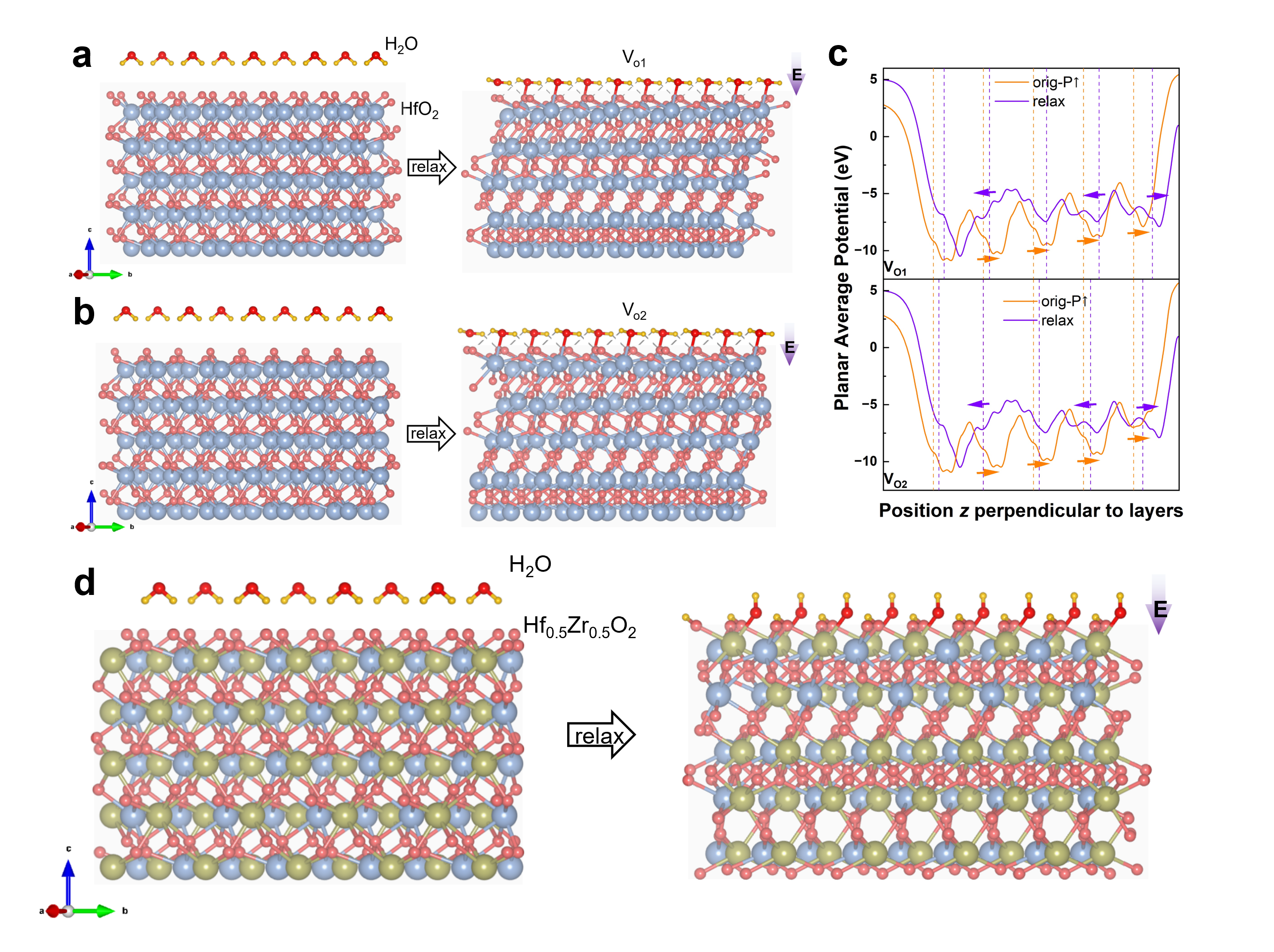


**Figure S9.** **Polarization directions affected by water molecules adsorption with oxygen vacancies at the surface.** (a, b) Schematic diagram of water molecule adsorption on the surface of ferroelectric orthorhombic hafnium oxide structure with two types of oxygen vacancies at the surface. The purple arrows represent the direction of the extra electric field generated by the water molecules. (c) The planar average of the electrostatic potential energy along the z direction with two types of oxygen vacancies at the surface. (d) Schematic diagram of water molecule adsorption on the surface of ferroelectric orthorhombic HZO structure with oxygen vacancy at the surface. The purple arrows represent the direction of the extra electric field generated by the water molecules.


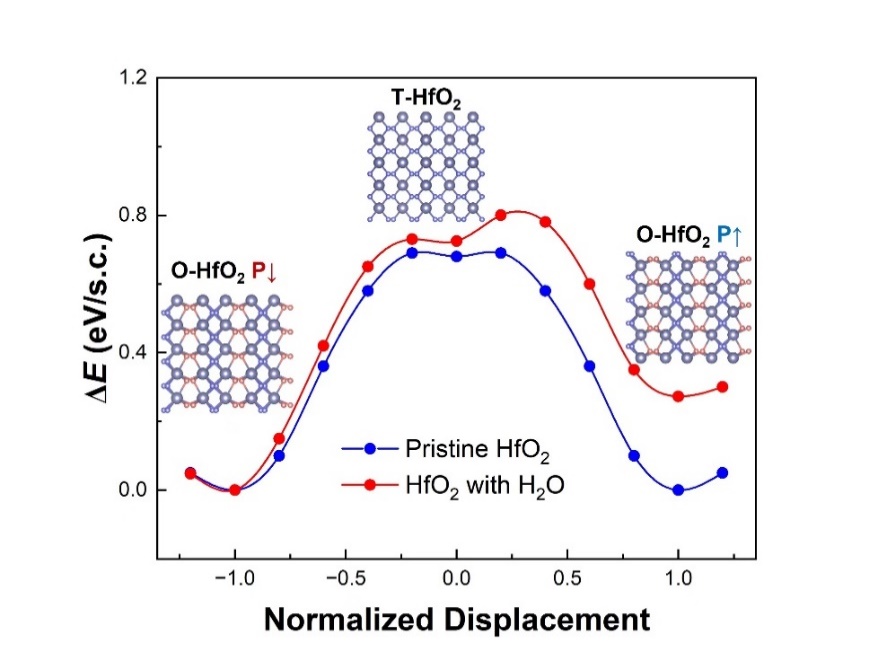


**Figure S10.** **DFT calculations for total energy profile as a function of normalized.** The ferroelectric orthorhombic phase with upward (downward) polarization is defined as +1, -1, 0 corresponds to the paraelectric tetragonal phase.


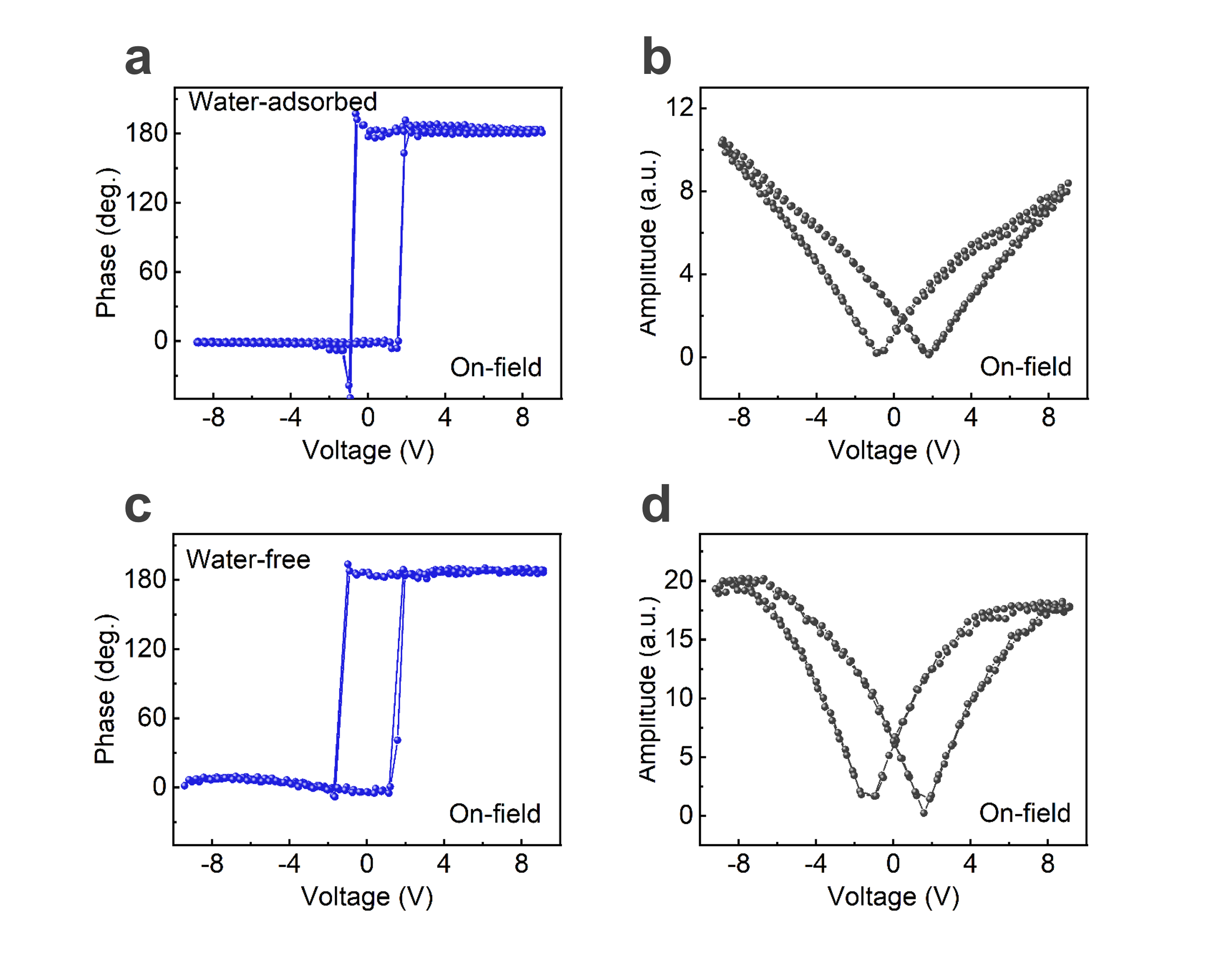


**Figure S11. On-field PFM loops for the graphene/(water)/HZO/silicon.** (a-d) The PFM hysteresis loop (a, c) obtained and amplitude butterfly (b, d) curves using on-field PFM method for Figure 4e, f.

**Note 1. PUND measurement**

First, an isotropic voltage greater than the coercive field is applied for pre-polarization, and then two equal pulses of voltage, i.e., "P" and "U", are applied. The current obtained by the first pulse "P" has a mixture of ferroelectric overturning current and non-ferroelectric contributions such as leakage and capacitance, while the second pulse "U" has only non-ferroelectric contributions due to the isotropic polarization and applied voltage. Subtracting the currents obtained from the two pulses gives a pure polarization flip-flop current and a true polarization value, as is the case for "N" and "D".

**Note 2. Experimental details**

**1. The ambient and high vacuum.** The ambient environment with the temperature of 23-25 °C, the relative humidity of 21-23%, and the air pressure of 101 kPa.

**2. The relative humidity.** The different relative humidity environments were achieved by the preparation of saturated salt solutions. For experimental environments with relative humidity of 24%, 55%, 72%, and 88% using saturated potassium acetate, magnesium nitrate, sodium chloride and potassium chloride solutions, respectively.

**Note 3. Principle of EFM**

EFM is based on the fact that van der Waals force and electrostatic force have different dominant mechanisms for detecting surface electrical signals, with van der Waals force proportional to 1/r^6^ and electrostatic force proportional to 1/r^2^. As shown in Figure S5, during the two scans of the EFM, the first scan (Tapping mode) is to get the topographic height image of the surface, and the second scan (Lift mode) lifts up a certain height and applies a certain bias voltage to the tip of the needle (AC + DC), and outputs the EFM phase signal along the first scanning trajectory. Attractive forces (gradients) between the probe and the sample surface increase the phase and repulsive forces (gradients) decrease the phase (Figure S5). The surface charge state is reflected by the variation of the EFM phase in different regions.

**Note 4. Details of theoretical analysis**

Due to the presence of electric polarization, band edges gradually shift layer-by-layer, eventually making the system metallic. Berry phase method and adiabatic Born effective charge approach are both inefficient for estimating polarization in this case. The electrostatic potential energy is thus adopted to identify the direction of depolarizing fields.

A global depolarizing field pointing to the left (right) can be clearly observed in the lower panel of Fig.3e (the upper panel of Fig.3e and Fig.S8c) with the Hf (O)-terminated surface, in consistent with surface charge conditions. Two polarization states of HfO_2_ are, in principle, energetically comparable. The presence of the global depolarizing field yields a preferred polarization in the same direction as this field. Independently, the local dipole moment can be determined by the potential walls on both sides of the planes containing Hf cations. For the HfO_2_-orig-P↑ cases (the blue line in Fig.3e and orange lines in Fig.S8c) as examples, the left potential walls are deeper in energy than those of the right ones, which is indicative of depolarizing fields pointing to the right, and thus the leftward electric dipoles. In dependent of the surface charge and polarization directions of HfO_2_, local dipole moments dominantly turn to the left in all situations after fully relaxing. Since the downward electric field generated by O-H bonds should additionally compensate for the depolarizing field caused by surface charge, its effect on tuning electric dipole moments would be slightly weaker in the Hf-terminated case. This is supported by the reduced energy differences between potential walls on both sides of the dotted lines in the lower panel of Fig. 3e. Our calculations for HfO_2_ slab with oxygen vacancies at the surface (Fig. S8) confirm that the considerable effect of water in air on the downward polarization of the HfO_2_ slab should be robust.
